# IGFBP-3 Blocks Hyaluronan-CD44 Signaling, Leading to Increased Acetylcholinesterase Levels in A549 Cell Media and Apoptosis in a p53-Dependent Manner

**DOI:** 10.1038/s41598-020-61743-3

**Published:** 2020-03-19

**Authors:** Deanna Price, Robert Muterspaugh, Bradley Clegg, Asana Williams, Alexis Stephens, Jeffrey Guthrie, Deborah Heyl, Hedeel Guy Evans

**Affiliations:** Chemistry Department, Eastern Michigan University, Ypsilanti, Michigan 48197 United States

**Keywords:** Cancer, Cell biology

## Abstract

Insulin-like growth factor binding protein-3 (IGFBP-3) belongs to a family of six IGF binding proteins. We previously found that IGFBP-3 exerts its cytotoxic effects on A549 (p53 wild-type) cell survival through a mechanism that depends on hyaluronan-CD44 interactions. To shed light on the mechanism employed, we used CD44-negative normal human lung cells (HFL1), A549, and H1299 (p53-null) lung cancer cells. A synthetic IGFBP-3 peptide (^215^-KKGFYKKKQCRPSKGRKR-^232^) but not the mutant (K228AR230A), was able to bind hyaluronan more efficiently than the analogous sequences from the other IGFBPs. In a manner comparable to that of the IGFBP-3 protein, the peptide blocked hyaluronan-CD44 signaling, and more effectively inhibited viability of A549 cells than viability of either H1299 or HFL1 cell lines. Treatment with the IGFBP-3 protein or its peptide resulted in increased acetylcholinesterase concentration and activity in the A549 cell media but not in the media of either HFL1 or H1299, an effect that correlated with increased apoptosis and decreased cell viability. These effects were diminished upon the same treatment of A549 cells transfected with either p53 siRNA or acetylcholinesterase siRNA. Taken together, our results show that IGFBP-3 or its peptide blocks hyaluronan-CD44 signaling via a mechanism that depends on both p53 and acetylcholinesterase.

## Introduction

Lung cancer is a devastating human disease and among the most common causes of cancer deaths worldwide^1,2^. Of all cases of the disease, non–small cell lung cancer (NSCLC) accounts for approximately 85%^3^.

CD44 is a type 1 transmembrane cell-surface glycoprotein with tumor promoting functions in many types of cancer cells^4–7^. It is the main cell surface receptor for hyaluronan (HA)^5–9^. Found on the extracellular side of the cell membrane is the CD44 globular HA-binding domain (HABD)^9,10^ shown previously to bind HA as a globular water-soluble protein^11^. CD44 is encoded by a single gene^5,6,12^ and many different variant isoforms (CD44v) are generated by alternative splicing that yield different patterns of amino acid insertion into the stalk domain of CD44 with the smallest being the standard CD44 (CD44s)^5,13–15^. Residues 32–123 in the N-terminal domain of CD44, common to both CD44s and CD44v isoforms, contain the HA-binding motif^16^. Assessment of CD44 expression in human lung cancer cell lines^17^, including A549 and H1299 used in this study, showed that the predominant isoform expressed is CD44s^18^. Being a common marker for tumor-initiating cells/cancer stem cells in human carcinomas, CD44 has gained much attention in the cancer literature^14^. HA-CD44 binding is known to modulate numerous downstream signaling cascades, such as the ERK1/2/MAPK and PI3K/Akt pathways, leading to tumor cell proliferation, survival, chemoresistance, and invasiveness^5,7,12,19^.

HA is a non-sulfated, anionic glycosaminoglycan^5,16,20,21^ polymer composed of the disaccharide sequence (D-glucuronic acid and D-N-acetylglucosamine) without known post-synthetic modification^6,22–24^. It is mostly abundant extracellularly and synthesized by HA synthases (HAS) localized at the cell membrane^5,7,19^. As a chief component of the extracellular matrix (ECM) and through interactions with its binding proteins, HA has been found to be implicated in the rapid remodeling of the matrix known to occur during the pathogenesis of many human diseases^19,25,26^. Binding of HA to CD44, its main receptor, is thought to vary in affinity^21,26–29^, promoting cell survival pathways^13^. Production and accumulation of HA in the tumor parenchyma is characteristic of certain cancers such as lung cancer and is associated with poor clinical outcomes^30^. The HAS inhibitor, 4-methylumbelliferone (4-MU)^31^, which does not alter the ability of CD44 to bind HA^32^, depletes glucuronic acid, a building block of HA synthesis and decreases expression of HAS2/3, by about 60–80% in cancer cell lines. Administration of 4-MU results in inhibition of downstream signaling mediated by HA with a consequent reduction in proliferation of cancer cells^6,30,33^.

Insulin-like growth factor binding protein 3 (IGFBP-3) belongs to a family of six IGF binding proteins that have highly conserved structures^34–39^. Acting as the main carrier of Insulin-like growth factor I (IGF-I) in the circulation and the most abundant IGFBP, IGFBP-3 can exert its antiproliferative functions by binding IGF-1, attenuating IGF/IGF-IR interactions^34,37,39^. IGFBP-3 is also known to regulate cell survival independently of the IGF/IGF-IR axis^39–42^. Expression of IGFBP-3 is reduced^43^ in lung cancer and associated with poor diagnosis in patients with stage I NSCLC^44–48^. There is an inverse relationship between plasma or serum levels of the protein and lung cancer risk^34,39,49^. Expression of IGFBP-3 led to increased cleaved caspase-3, inactivation of MAPK signaling, and corresponded with diminished survival of human lung cancer cells^50^. Recently, we found that IGFBP-3 binds HA through residues 215–232 in the C-terminal region of the protein (^215^-KKGFYKKKQCRPSKGRKR-^232^) and blocks its interactions with CD44, reducing cell viability of A549 human lung cancer cells^51^. These results are consistent with previous reports showing that this region of IGFBP-3 is able to bind certain glycosaminoglycans including HA^34,39,52–54^. We also showed that blocking HA-CD44 binding with an anti-CD44 antibody (5F12), known to be antagonistic towards HA-CD44 molecular interactions in combination with IGFBP-3, did not have an additive negative effect on cell viability, suggesting that IGFBP-3 exerts its cytotoxic effects on cell survival through a mechanism that depends on HA-CD44 interactions^51^. Here, we aim to provide a clearer picture of the mechanism by which blocking HA-CD44 interactions with IGFBP-3, in the absence or presence of the anti-CD44 antibody or 4-MU, results in diminished cell survival.

In response to various cellular stresses, the p53 tumor suppressor protein regulates the expression of a large number of genes involved in inhibition of cell proliferation, cell-cycle arrest, induction of apoptosis and senescence^55,56^. P53 has been previously shown to inhibit expression of the CD44 cell-surface protein by binding to a noncanonical p53-binding sequence in the CD44 promoter^15,57^. P53, operating in lung carcinoma cells, was found to directly influence the promoter of the CD44 gene, acting as a repressor of CD44 protein expression^57^. In cells lacking p53 function, de-repression of CD44 led to the survival of tumor growth, anti-apoptotic and mitogenic effects^57^. In hepatocellular carcinoma, CD44 was shown to induce AKT activation, which in turn resulted in phosphorylation and translocation of Mdm2, a negative regulator of p53, to the nucleus, terminating the p53 response^58^. High CD44 expression can promote growth and survival in different stages of tumor progression by counteracting p53 tumor-suppressor function while p53 acts to repress CD44 expression to promote its apoptotic and antiproliferative activities^57,58^.

P53 action was shown previously to be blocked by antagonizing IGFBP-3, a p53-response gene that mediates p53-induced apoptosis during serum starvation in an IGF-independent manner in cancer cells^59^. P53 induces IGFBP-3 expression and targeting p53 for degradation in lung carcinoma H460 cells resulted in decreased apoptosis and enhanced cell growth during serum deprivation compared to untreated cells^59^.

A downstream component of p53 was found to be acetylcholinesterase (AChE) in MCF-7 cells treated with cisplatin, an anti-tumor drug^60,61^. Upregulation of AChE expression was observed in response to activation of p53 in apoptotic MCF-7 cells treated with cisplatin, while this cisplatin-induced AChE expression was abolished when p53 expression was blocked with siRNA^60^. Therefore, AChE might be a downstream component of p53 that leads to induction of apoptosis in chemotherapy^60,61^.

AChE, encoded by a single gene, is a member of the serine hydrolase family using a serine residue at the active site^62^. While AChE is well-known for its classical key role in the catalytic hydrolysis of cholinergic neurotransmitters, recent studies have shown non-classical functions of the enzyme as a potentially promising tumor growth suppresser and regulator of apoptosis^61^, suggesting that elevated AChE expression and/or activity in response to apoptotic stimuli could serve as a marker of apoptosis and promising anticancer therapeutic^63,64^. While cells overexpressing AChE undergo apoptosis more easily than controls, AChE is not thought to be an apoptosis initiator^64^. Some tumor cells show no expression of AChE and are not sensitive to apoptosis induction suggesting that low levels of AChE protect the cells against apoptosis^61,63,64^.

Acetylcholine is known to be secreted by lung cancer cells into the extracellular environment, stimulating growth of cancerous cells in lung tumors^65,66^. It acts as an autocrine growth factor for human lung cancer^67^ by binding to nicotinic and muscarinic receptors on lung cancer cells, accelerating their proliferation, migration, and invasion^67^. Acetylcholine was found to have mitogenic effects in A549 and in p53-negative human lung carcinoma NSCLC H1299 cells, increasing the expression of matrix metalloproteinases and downregulating the expression of E-cadherin in A549 human NSCLC^61,67^.

While AChE is not an apoptosis initiator, it acts as a tumor suppressor, in part by the catalytic hydrolysis of acetylcholine^61,68–70^. The activity of AChE was found to be reduced in lung cancer, likely contributing to increased acetylcholine levels, lung cancer growth and tumor aggressiveness, poor prognosis, and low survival rate^61,68–70^. Apoptosis was prevented when using anti-sense oligonucleotides of AChE^71^. In addition, while the expression level of AChE increases during apoptosis in different cell types, blocking AChE expression by siRNA or pharmacological inhibition of AChE prevented apoptosis^61,71^. No AChE expression was detected in the normal human lung fibroblast (HLF) cells until the cells were treated to undergo apoptosis^61^. AChE may function as a pro-apoptotic gene in NSCLC cells, and attenuates cell growth when its expression is upregulated^61,69^.

In this study, we show that blocking HA-CD44 interaction with IGFBP-3 results in increased levels of active AChE in the media of A549 cells, acting as an active participant in apoptosis in a p53-dependent and IGF-independent fashion.

## Results

### A peptide corresponding to residues 215–232 of IGFBP-3, but not the corresponding mutant peptide (K228AR230A), binds HA

**Figure 1:**
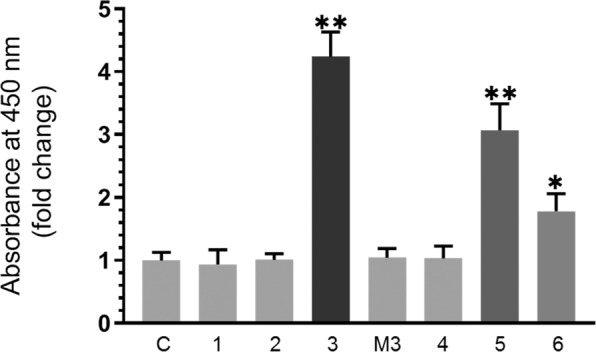
The IGFBP-3 peptide, but not its mutant, binds biotinylated HA. Each peptide (50 nM) was allowed to bind to ELISA wells overnight. The wells were blocked, then incubated with 200 nM biotinylated-HA for 24 h and processed as described in the text. Optical density measurements were normalized by expressing each point in relation to the control (C) using BSA alone and fold change relative to the control was calculated. Each column represents the mean ± S.D. of three independent experiments, each run in triplicate and plotted using the GraphPad Prism 8.3.1 software. The asterisks (*p < 0.05, **p < 0.0l) indicate a statistically significant difference from the control. Absence of asterisks indicates no significance, Mann-Whitney test.

Previously^51^, we found that the IGFBP-3 protein and a synthetic IGFBP-3 peptide (^215^-KKGFYKKKQCRPSKGRKR-^232^), derived from the carboxy-terminal region of the protein, bind HA and block its ability to bind CD44. Inspection of residues 215–232 shows two overlapping sequences (Table 1, first and last residues of the sequences shaded) that contain the [B(X_7_)B] motif previously reported to be necessary to bind HA, where “B” can be either arginine or lysine, and “X” any non-acidic amino acid^9^. To synthesize an IGFBP-3 peptide that lacks the ability to bind HA, we constructed a mutant (K228AR230A) that disrupts the HA-binding motif (Table 1). In addition, we synthesized the corresponding peptides of IGFBP 1–6 (Table 1) to examine whether the analogous sequences in other IGFBPs are also able to bind HA. Each peptide (50 nM) was bound to ELISA wells overnight. After blocking, the wells were then incubated with 200 nM biotinylated-HA for 24 h and processed as described in the Methods section. Optical density measurements were normalized by expressing each point in relation to the control (C) using BSA alone and fold change relative to the control was calculated. Each column represents the mean ± standard deviation (S.D.) of three independent experiments, each performed in triplicate and plotted using the GraphPad Prism 8.3.1 software (Fig. 1). The IGFBP-3 peptide was more efficient in binding HA. Albeit less efficiently, the IGFBP-5 and IGFBP-6 peptides also showed some binding to HA while no binding above control was observed for the IGFBP-3 mutant peptide or for IGFBP peptides 1, 2, or 4.

**Table 1:** Synthetic peptide sequences and molecular weights. The first and last amino acid residues of the overlapping HA binding sequences in IGFBP-3, are shaded.

IGFBP Peptides	Sequence	Molecular Weight (Da)
IGFBP-1	^183^KNGFYHSRQCETSMDGEA^200^	2057.9
IGFBP-2	^227^KHGLYNLKQCKMSLNGQR^244^	2116.1
IGFBP-3	^215^KKGFYKQCRPSGKR^232^	2221.4
IGFBP-4	^185^RNGNFHPKQCHPALDGQR^202^	2073.0
IGFBP-5	^201^RKGFYKRKQCKPSRGRKR^218^	2278.3
IGFBP-6	^168^HRGFYRKRQCRSSQGQRR^185^	2304.2
IGFBP-3 mutant, M3 (K228AR230A)	^215^KKGFYKKKQCRPSAGAKR^232^	2079.2

### The IGFBP-3 protein, its wild-type but not the mutant peptide, block HA-CD44 signaling and diminish cell viability of the p53-positive A549 cell line more effectively than either the CD44-negative HFL1, or p53-negative H1299 cell lines

**Figure 2:**
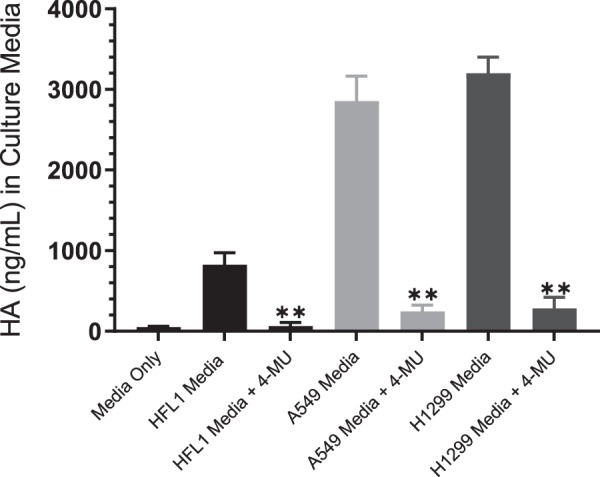
HA concentration in the culture media is reduced following treatment with 4-MU. Cells (0.2 × 10^5^) were grown in 10% FBS-supplemented media overnight then in serum-free medium for 12 h prior to treatment with 600 µM 4-MU. The cells were then incubated for 48 h and the media collected. Samples (3 µL of 600 µg/mL total protein) were assayed for HA using the hyaluronan quantikine ELISA kit according to the manufacturer’s recommendation (R&D Systems). Media not incubated with cells was used as a negative control. The graphs prepared using the GraphPad 8.3.1 software, summarize the results expressed as means ± S.D. of five independent experiments, each performed in triplicate. Asterisks (**) indicate a statistically significant difference from the corresponding untreated cell line control, **p < 0.0l of each cell line.

The effect of the IGFBP-3 protein and the IGFBP peptides (Table 1) on cell viability was examined in a normal human lung cell (HFL1) reported earlier^72^ to be CD44-negative and two human NSCLC cell lines^43^, A549 expressing relatively high levels of IGFBP-3 and p53, and H1299 with undetectable levels of IGFBP-3 and a p53-null genotype due to a biallelic deletion of the TP53 gene^73^. Cells were seeded in 96-well plates at 0.2 × 10^5^ cells per well in 10% FBS-supplemented media. The following day, the cell monolayers were incubated in serum-free medium for 12 h, then treated as indicated for 48 h either with 600 µM 4-MU or with the media containing the specific components in the different treatments replaced every 12 h. Samples (3 µL of 600 µg/mL total protein) were assayed for HA levels (Fig. 2) using the hyaluronan quantikine ELISA kit according to the manufacturer’s recommendation (R&D Systems). The medium not incubated with cells was used as a negative control. Measurement of the HA concentration accumulated in the culture media showed that A549 and H1299 cells secreted a 3–4 fold higher level of HA as compared to the amount secreted by HFL1 cells (Fig. 2). Moreover, following treatment with 4-MU, HA synthesis was significantly decreased in all cell lines compared with the control. The IGFBP-3 protein or peptides (50 nM) were added to cells in the absence or presence of 600 µM 4-MU or 5 μg/mL of the CD44 antibody, 5F12, known to block HA-CD44 interactions^4,74^. This concentration of added IGFBP-3 was chosen to be close to the values we measured previously in the conditioned media of A549 cells^51^. When added in combination, 4-MU or the CD44 antibody were added 24 h or 2 h prior to addition of IGFBP-3 protein or peptides, respectively. Cell viability was assessed by the MTT assay as described in the Methods section. Optical density measurements (570 nm) were normalized by expressing each point in relation to the untreated control of each cell line (set to 100%). Each column represents the mean ± S.D. of three independent experiments. Treatment of HFL1 cells with either 5F12 or 4-MU had little effect on cell viability (Fig. 3A). This was not surprising since HFL1 have been shown to express very little or no CD44 receptor^72^. Only a small effect was observed upon adding the IGFBP-3 protein alone or in combination with the antibody, 5F12, or 4-MU treatment. In accord with our previous results^51^, we found that blocking HA-CD44 interactions in A549 cells with 5F12 reduced cell viability to the same extent as that found by the addition of only IGFBP-3 (Fig. 3B). Moreover, the negative effects of both treatments were not additive indicating that IGFBP-3 reduces cell viability by disrupting HA-CD44 interactions. Similarly, reduction of cell viability upon pretreatment of A549 cells with the HAS inhibitor, 4-MU, was not further augmented by the addition of the IGFBP-3 protein. While similar effects were observed in the p53-negative cell line, H1299 (Fig. 3C), the inhibition of cell viability was only 10–25% as compared to 50–65% observed in A549 cells. These results suggest that H1299 might be more resistant to the effects of blocking HA-CD44 with 5F12, 4-MU, or IGFBP-3. Since we found earlier^51^ that both the IGFBP-3 protein and its peptide bind HA with comparable affinity, we examined the effect of the wild-type (WT) peptide on cell viability relative to the full-length protein (Fig. 3D). Not surprisingly, the effects on cell viability were comparable suggesting that the mature IGFBP-3 protein exerts its effect on HA-CD44 signaling via residues 215–232 of the protein. The effects of the mutant IGFBP-3 peptide (Fig. 3E) on HFL1 were comparable to those of the full length IGFBP-3 protein and WT peptide (Fig. 3A,D). This is likely due to the lack of CD44 expression in these cells. Incubation of A549 or H1299 with the mutant peptide alone (Fig. 3E), had little effect on cell viability compared to the IGFBP-3 protein (Fig. 3B,C) or the WT peptide (Fig. 3D) suggesting that binding HA is a prerequisite for the IGFBP-3 peptide to exert its effects on cell viability. Moreover, the heparin-binding sequence [B-B-B-X-X-B], where B is a basic residue, arginine, lysine, or histidine, and X is any residue, found within the IGFBP-3, -5 and -6 basic peptide sequences, is maintained in the IGFBP-3 mutant, indicating that this region is not necessary for either binding HA or induced effects on cell viability.

**Figure 3(A–E):**
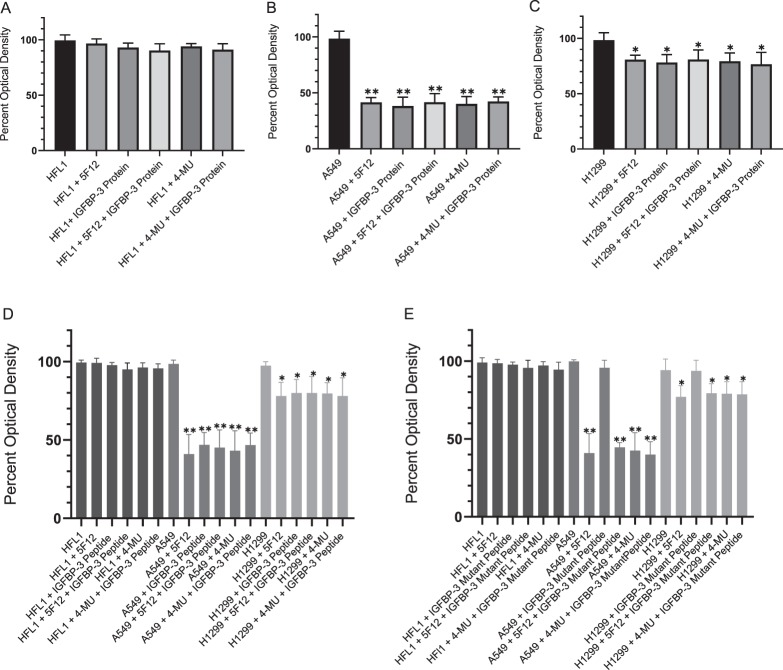
The IGFBP-3 protein and WT peptide, but not the mutant, block HA-CD44 signaling and more effectively inhibit cell viability of A549 cells than either the p53-negative H1299 or CD44-negative HFL1 cell lines. IGFBP-3 protein or peptides were added to cells in the absence or presence of the CD44 antibody, 5F12, known to block HA-CD44 interactions, or 4-MU. Cell viability was assessed by the MTT assay. Cells were seeded in 96-well plates at 0.2 × 10^5^ cells per well in 10% FBS-supplemented media. The following day, the cell monolayers were incubated in serum-free medium for 12h, then treated as indicated for 48h with the media containing the specific components in the different treatments replaced every 12h. The concentration of IGFBP-3 protein or peptides added was 50 nM and that of 4-MU was 600 μM. The CD44 antibody (5 μg/mL) was added either separately or 2h prior to addition of IGFBP-3 and/or peptides. Optical density measurements (570 nm) were normalized by expressing each point in relation to the untreated control of each cell line (set to 100%). Each column represents the mean ± S.D. of three independent experiments, each run in triplicate. Asterisks (*) indicate a statistically significant difference from the corresponding untreated cell line control, *p<0.05, ** p<0.0l of each cell line. Absence of asterisks indicates no significance, Mann-Whitney test.

**Figure 3(F–J):**
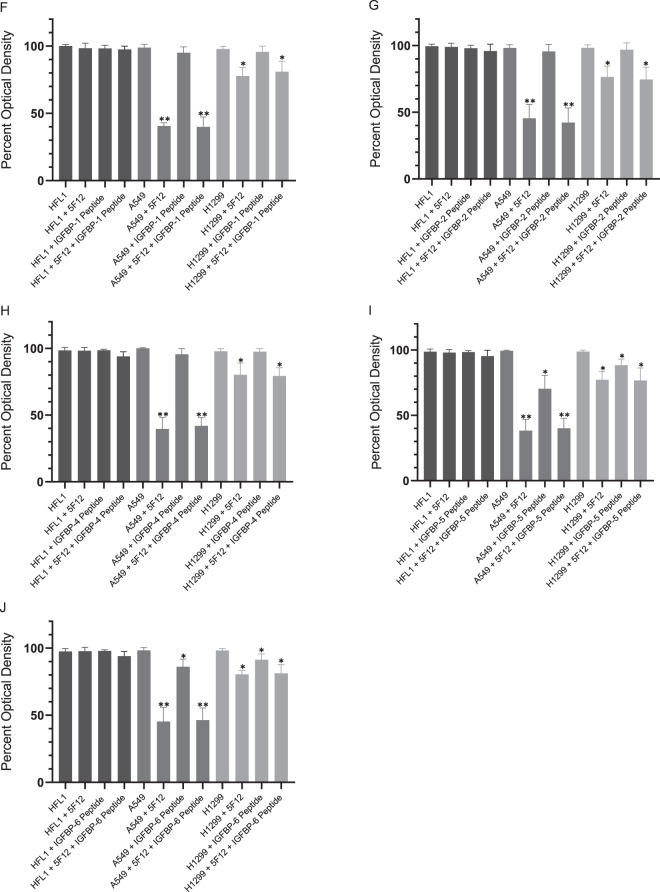
The IGFBP-5 and IGFBP-6 peptides are able to block HA-CD44 signaling and cell viability of A549 cells, albeit less effectively than the IGFBP-3 peptide. Cell viability was measured as described in (**A–E**) legend.

### The IGFBP-5 and IGFBP-6 peptides are less effective at reducing cell viability than the IGFBP-3 peptide

The proteins IGFBP-1 to −6 share similar structural organization consisting of three distinct N-terminal, linker, and C-terminal domains^34,38^. Besides their IGF-dependent roles, almost all the IGFBPs have IGF-independent functions^75,76^ that are less understood and are due in part to their ability to interact with ECM components. Neither IGFBP-1 nor IGFBP-4 peptides (Fig. 3F,H) had any effect on viability of any of the cell lines examined. We did not expect an effect on HFL1 since it is CD44-negative. The lack of an effect on A549 or H1299 is likely due to the absence of the HA binding motif [B(X_7_)B] found in the IGFBP-3 and −5 peptides. Similarly, no effect on cell viability upon addition of the IGFBP-2 peptide was observed (Fig. 3G). Residues important for binding heparin have been previously identified in IGFBP-2 by site-directed mutagenesis or NMR that encompass Lys227, His228, Asn232, Leu233, Lys234 and overlap the analogous IGFBP-3 heparin-binding region^34,36,38^. Both the lack of binding to HA (Fig. 1) and the lack of an effect on cell viability (Fig. 3G) suggest that these residues are not important in HA-dependent signaling.

Heparin binding motifs have been shown to be located within the C-terminal domains of IGFBP-3, −5, and −6 proteins, and for IGFBP-3 and −5 function in binding to the cell surface and/or the ECM^34,36,38^. Like the IGFBP-3 peptide, both IGFBP-5 and IGFBP-6 peptides reduced cell viability of A549 more effectively than that of H1299 whether the peptides were added separately or in combination with the 5F12 antibody (Fig. 3I,J). The extent of the inhibition correlated with their efficiency of binding to HA (Fig. 1) and was less pronounced compared to that found for the IGFBP-3 peptide. No effect on cell viability was found for IGFBP-3, −5, or -6 on HFL1 cells which is not surprising since these cells are deficient in CD44. These results suggest that the IGFBP-5 and -6 peptides might also work by disrupting HA-CD44 signaling, albeit with lower efficiency than the IGFBP-3 peptide, and that this effect is more pronounced in the p53-positive cell line, A549.

We previously showed that the IGFBP-3 peptide exhibits similar binding affinities to HA as the full-length protein^51^. The results here show that the IGFBP-3 peptide is better able to bind HA than either IGFBP-5 or −6 peptides (Fig. 1) and that its effects on cell viability are more comparable to those of the IGFBP-3 protein than the other peptides (Fig. 3). For those reasons and to further shed light on a mechanism by which IGFBP-3 disrupts cell viability more efficiently in A549 cells than H1299, we chose to focus on the IGFBP-3 protein, the IGFBP-3 peptide, and its mutant as a negative control for the rest of the experiments in this work.

### Cell treatment with either the IGFBP-3 protein, the CD44 antibody, 4-MU, or in combination, results in increased levels and activity of AChE in the media of A549 cells but not in the media of either HFL1 or H1299

**Figure 4:**
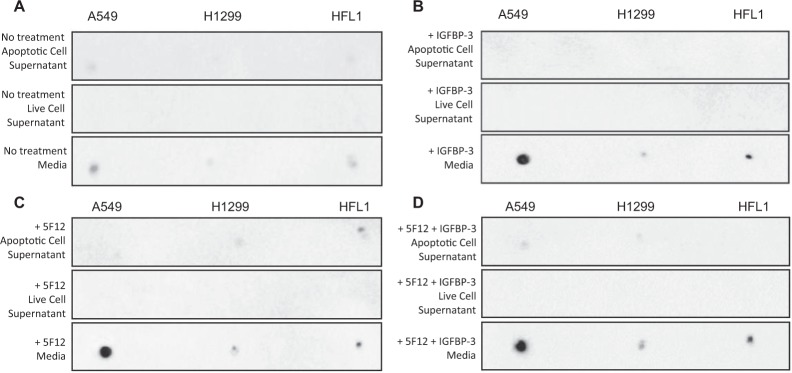
AChE concentration is increased to a comparable extent upon treatment with the IGFBP-3 protein, 5F12, or in combination, in the media of A549 cells but not in the media of the CD44-negative cell line, HFL1, or the p53-negative cell line, H1299. Cells (0.2 × 10^5^) were grown in 10% FBS-supplemented media overnight then the cell monolayers were incubated in serum-free medium for 12 h. The cells were then treated with 50 nM IGFBP-3 protein, 5 μg/mL anti-CD44 antibody (5F12), or in combination. The cells were then allowed to incubate for 48 h and the media collected. Attached live cells and detached apoptotic cells were harvested and the cell pellets were resuspended in 1 mL lysis buffer (Methods). The same amount of protein (3 µL of 600 µg/mL total protein) of the samples were spotted onto a nitrocellulose membrane. The blots were incubated with goat anti-AChE antibodies and the amount of AChE on the membrane was detected using super signal west pico luminol (chemiluminescence) reagent, imaged with a Bio-Rad molecular imager, and quantitated using Image J (Methods).

**Figure 5:**
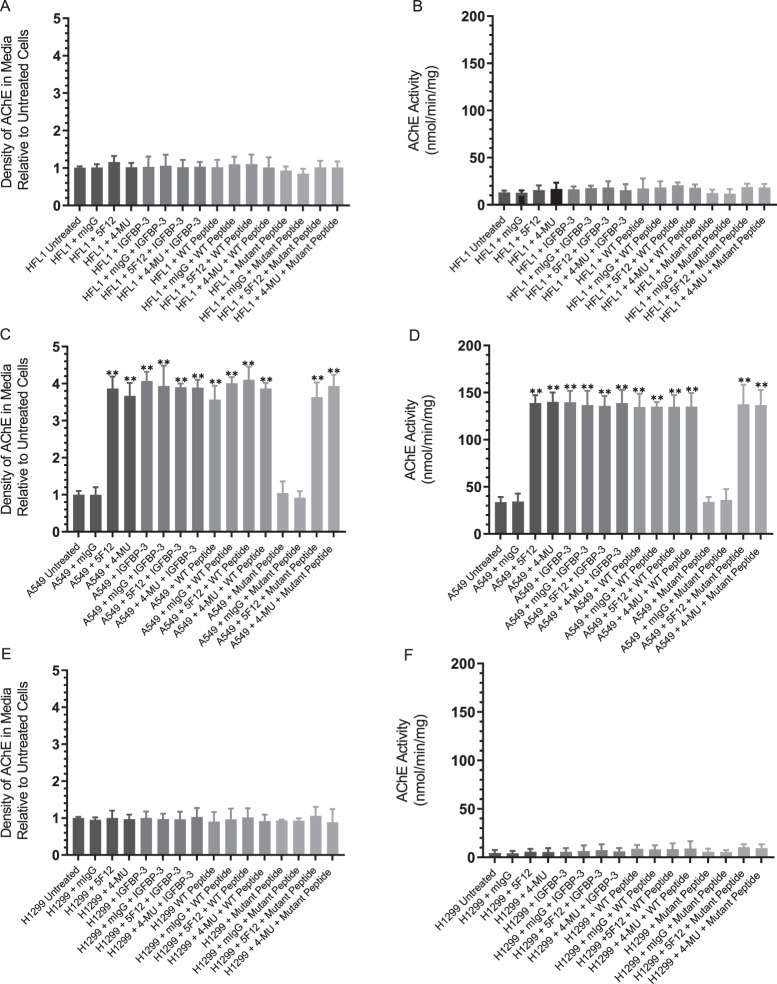
Both AChE concentration and activity are increased to a comparable extent in the media of A549 cells upon treatment with the IGFBP-3 protein or peptide, 5F12, 4-MU, or in combination. Cells (0.2 × 10^5^) were grown in 10% FBS-supplemented media overnight then in serum-free medium for 12 h prior to treatment with mIgG (5 μg/mL), 5F12 (5 μg/mL), 50 nM IGFBP-3 protein or peptides, 600 µM 4-MU, or in combination. The cells were then incubated for 48 h and the media collected. Samples (3 µL of 600 µg/mL total protein) were spotted onto a nitrocellulose membrane and AChE was visualized using anti-AChE antibodies (Methods). The dots were quantitated, averaged, normalized and expressed as fold change relative to untreated cells (**A,C,E**). AChE activity using the same amount of protein was measured as described in Methods (**B,D,F**). The graphs prepared using the GraphPad 8.3.1 software, summarize the results expressed as means ± S.D. of five independent experiments, each performed in triplicate. Asterisks (*) indicate a statistically significant difference from the corresponding untreated cell line control, *p < 0.05, **p < 0.0 l of each cell line. Absence of asterisks indicates no significance, Mann-Whitney test.

Both the IGFBP-3 protein and WT peptide, but not the mutant, were more effective at reducing cell viability of the p53-positive A549 cell line as compared to the p53-negative cell line, H1299 (Fig. 3A–E). It was found earlier that AChE is a downstream component of p53 in MCF-7 cells treated with the anti-tumor drug, cisplatin, raising the possibility that AChE expression could be upregulated by p53 activation during the induction of apoptosis^60,61^. In addition, the expression level of AChE was found to be upregulated during apoptosis in different cell types^61,71^ including NSCLC cells, attenuating cell growth^61,69^. We, therefore, tested the amount of AChE in the supernatant and media of cells treated with the IGFBP-3 protein (50 nM), the 5F12 antibody (5 μg/mL), and in combination where the CD44 antibody was added 2 h prior to addition of IGFBP-3 (Figs. 4, 5). After cell treatments (Methods), the protein concentrations of the media and cell supernatants were determined using the BCA protein assay kit. Total protein (3 µL of 600 µg/mL) of supernatants prepared from live attached cells or from detached apoptotic cells, and from the conditioned media were spotted onto a nitrocellulose membrane. The blots were incubated with goat anti-AChE antibodies and the amount of AChE on the membrane was visualized. Untreated cells (Fig. 4A) showed basal level of AChE in both the apoptotic cell supernatants and the media but not in the live cell supernatants. Treatment of the cells with IGFBP-3 showed an approximate 4-fold increase (Figs. 4B,5C) in the amount of AChE in the media of A549 cells but not in the media of either HFL1 or H1299 (Figs. 4B, 5A,E). Similar results were obtained upon treating the cells with only the 5F12 antibody that is known to block HA-CD44 signaling, the HAS inhibitor, 4-MU, (Figs. 4C, 5A,C,E) or in combination with IGFBP-3 (Figs. 4D, 5A,C,E), suggesting that inhibiting HA synthesis or blocking HA-CD44 interaction with either the antibody or with IGFBP-3, increases the levels of AChE in the media of A549 cells.

The mean AChE activity in the healthy lung was shown earlier to be 10.95 ± 6.90 mU/mg^67,77^. Since the increase in the levels of AChE was found in the media (Fig. 4) of A549 cells, the treatments were repeated on all three cell lines and both the levels and activity of the protein in the media were determined. The graphs (Fig. 5) summarize the results expressed as the mean ± S.D. of five independent experiments, each run in triplicate. As expected, treatment of the cells with mouse IgG isotype control with no relevant specificity to a target antigen (mIgG, 5 μg/mL) had no effect on the level or activity of AChE compared to untreated controls in any of the three cell lines. Addition of 5 μg/mL anti-CD44 antibody (5F12) known to be antagonistic towards HA-CD44 molecular interactions or treatment with 600 µM 4-MU, resulted in an approximate 4-fold increase in the level and activity of AChE only in A549 cell media (Fig. 5CD). The lack of response using HFL1 is not surprising since they are CD44-negative, however, the absence of an effect using the p53-negative H1299 cell line under these conditions despite the presence of CD44, might suggest a role for p53 in regulating the levels and activity of AChE upon disruption of HA-CD44 interactions. Addition of only 50 nM IGFBP-3 protein or WT but not the mutant peptide, resulted in a comparable increase in AChE levels and activity in the media of A549 cells. Incubation with 600 µM 4-MU for 24 h or the anti-CD44 antibody for 2 h prior to addition of either 50 nM IGFBP-3 protein or WT peptide did not result in an additive effect on the level or activity of AChE, suggesting that the IGFBP-3 protein or peptide exert their effect on AChE through a mechanism that depends on HA-CD44 interactions.

### Treatment with the IGFBP-3 protein or WT but not the mutant peptide of A549 cells transfected with either p53 siRNA or with AChE siRNA, results in decreased AChE levels and activity in the media, decreased apoptosis, and increased cell viability

**Figure 6:**
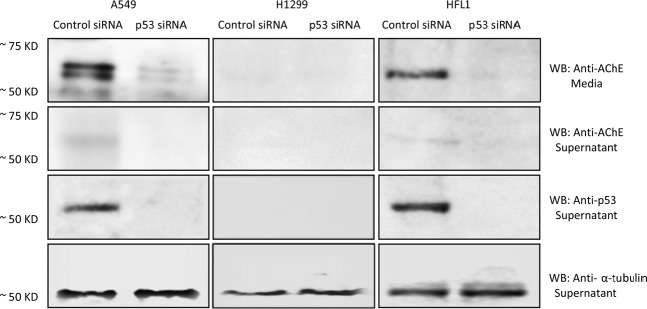
The levels of AChE are decreased in A549 cells transfected with p53 siRNA. Cells were seeded at a density of 2 × 10^4^ cells in 25 cm^2^ flasks. The following day, control siRNA or p53 siRNA was mixed with Lipofectamine 2000 transfection reagent (ThermoFisher) for 20 min at RT. The mixtures were then added to the cells to a final concentration of 100 nM for each siRNA. The cells were then allowed to incubate at 37 °C in serum-free media for 72 h. The same concentration of total protein (15 µL of 600 µg/mL) of the cell supernatants and media was used for Western blotting using the indicated antibodies.

Treatment with 100 nM p53 siRNA resulted in reduced expression of AChE in both A549 cell supernatant and media, results similar to those obtained for HFL1 (Fig. 6). While a single band for AChE was identified in HFL1 media, more than one band reacted with the antibody in the media from A549 cells. This observation might be due to the presence of different forms of the enzyme in A549 cells reported previously^70^ or glycosylation as has been previously observed in human breast cancer^78^. While as expected, no signal was obtained for p53 in the p53-negative cell line, H1299, the AChE levels were barely detectable in either the supernatant or media with cells transfected with control or p53 siRNA (Fig. 6). In support of previous findings^60^, these results suggest that expression of AChE might be regulated by p53.

**Figure 7:**
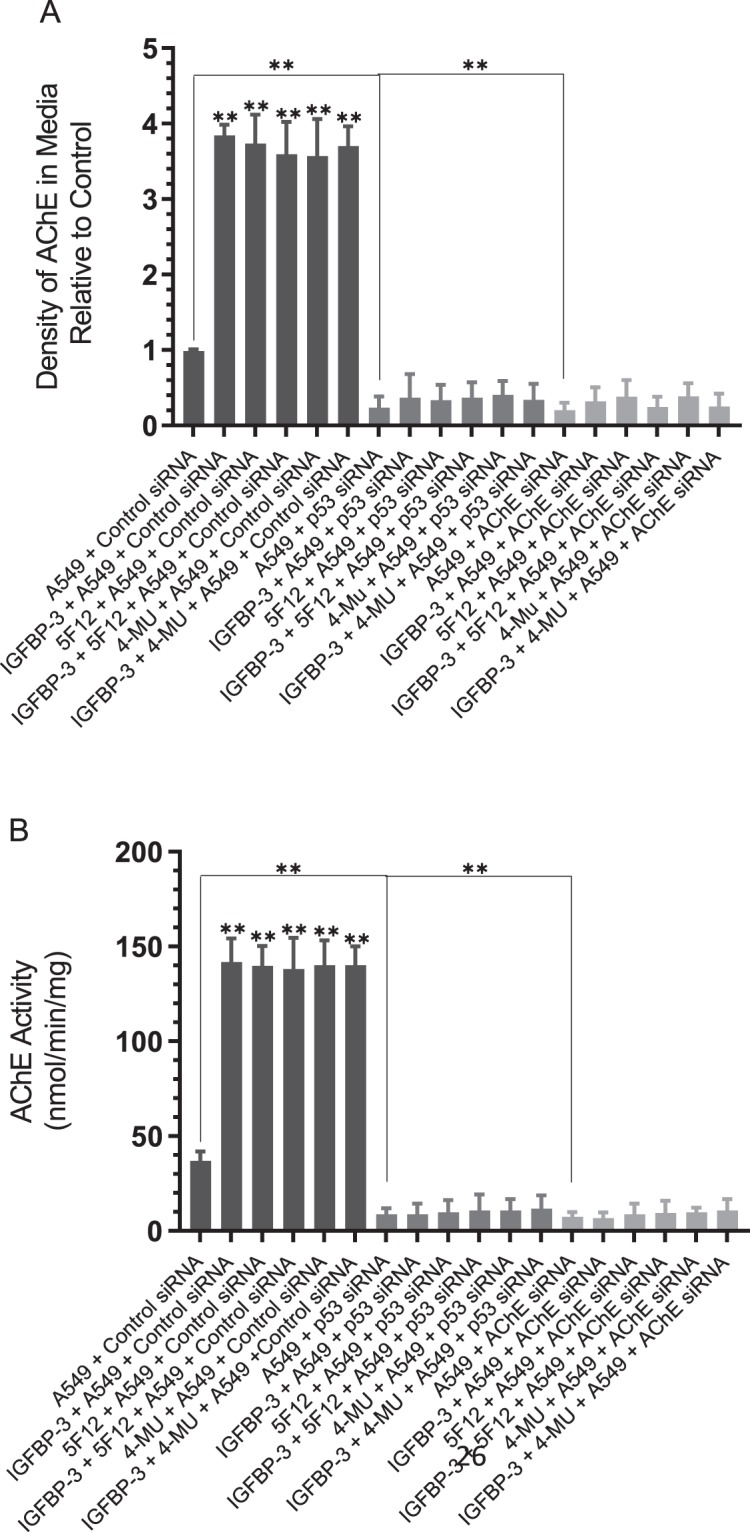
Both AChE concentration and activity are decreased in A549 cells transfected with either p53 siRNA or AChE siRNA and treated with IGFBP-3. (**A**) After cell treatments, 3 µL of 600 µg/mL total protein of the conditioned media were used to determine the amount of AChE. Samples were spotted onto a nitrocellulose membrane and AChE was visualized using anti-AChE antibodies (Methods). The dots were then quantitated using Image J, averaged, normalized and expressed as fold change relative to untreated cells. (**B**) AChE activity was measured as described in the Methods section. Processing of the data was carried out with the GraphPad 8.3.1 software. The graphs summarize the results expressed as means ± S.D. of three independent experiments, each performed in triplicate. Asterisks (**) indicate a statistically significant difference between each treatment relative to cells with siRNA only and between p53 or AChE siRNA treatment relative to control siRNA. Absence of asterisks indicates no significance, Mann-Whitney test, **p < 0.0 l.

Both expression (Fig. 7A) and activity (Fig. 7B) of AChE were diminished in A549 cell media upon transfection of the cells with p53 siRNA. Increased AChE levels and activity (~4-fold) were obtained upon treatment of A549 cells transfected with control siRNA, with either 50 nM IGFBP-3, 600 µM 4-MU, 5 μg/mL 5F12, or in combination (Fig. 7A,B), results consistent with those obtained in Figs. 4–5. These effects were abolished upon the same treatments of A549 cells transfected with p53 siRNA. Similar results were obtained with the WT but not the mutant peptide (Supplementary Table 2AB). No such increase was found in either HFL1 or H1299 (Supplementary Table 2AB) which is likely due to the lack of expression of CD44 and p53 in HFL1 and H1299, respectively. Moreover, relative to control cells transfected with p53 siRNA, no increase in the levels or activity of AChE in the media was observed in either H1299 or A549 cells transfected with p53 siRNA and treated with either IGFBP-3 protein, WT but not the mutant peptide, in the absence or presence of 5F12 or 4-MU (Fig. 7AB, Supplementary Table 2A,B). These results point to the importance of p53 as a key player in the mechanism employed by IGFBP-3 in regulating the levels and activity of AChE in the media.

**Figure 8:**
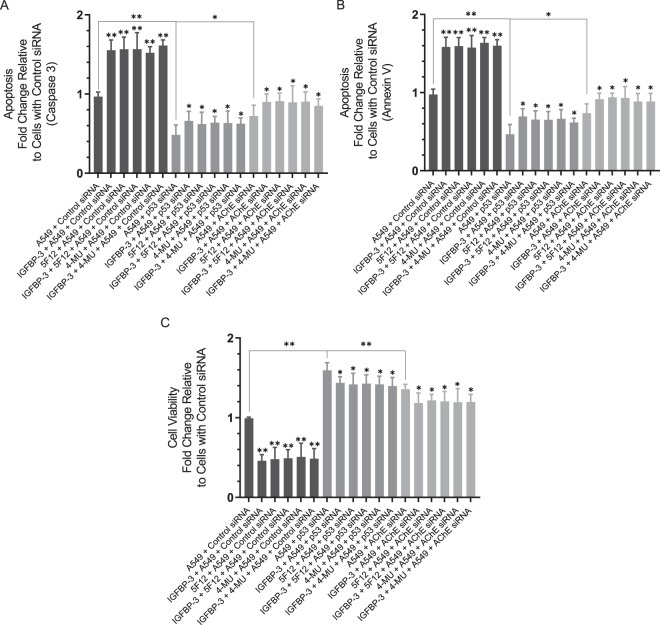
A549 cells transfected with either p53 siRNA or AChE siRNA and treated with IGFBP-3 exhibit diminished apoptosis which correlates with increased cell viability. When measuring apoptosis using the caspase 3 assay (**A**) or the annexin V method (**B**) or cell viability (**C**), cells (2 × 10^4^ cells/well in 200 µL medium) were plated in a 96-well plate. After 24 h of incubation with the siRNA, cells were treated for an additional 48 h, then apoptosis or cell viability were measured as described in Methods. Processing of the data was carried out using the GraphPad 8.3.1 software. The graphs summarize the results expressed as means ± S.D. of three independent experiments, each performed in triplicate. Asterisks (*) indicate a statistically significant difference between each treatment relative to cells with siRNA only and between p53 or AChE siRNA treatment relative to control siRNA. Absence of asterisks indicates no significance, Mann-Whitney test, *p < 0.05, **p < 0.0 l.

Inhibition of expression of either p53 or AChE using antisense oligonucleotides in A549 cells resulted in decreased apoptosis, measured by the cleaved caspase 3 (Fig. 8A) or the annexin V (Fig. 8B) method, and a corresponding increase in cell viability (Fig. 8C). Treatment of A549 cells transfected with control siRNA with the IGFBP-3 protein, WT but not the mutant-peptide, in the absence or presence of 5F12 or 4-MU (Fig. 8, Supplementary Tables 3AB and 4) resulted in increased apoptosis and decreased cell viability. While these effects were reduced upon the same treatments of cells transfected with either p53 or AChE siRNA, in all cases, there was a relative increase in apoptosis and a relative decrease in cell viability compared to cells treated with only siRNA (Fig. 8, Supplementary Tables 3AB and 4). These results suggest that while p53 and AChE are important players in the mechanism utilized by IGFBP-3, other pathways are likely employed by IGFBP-3, in addition.

To test the effect of inhibiting the catalytic activity of AChE on IGFBP-3 induced effects, treatment of the cells with 1 µM tacrine, a cholinesterase inhibitor, reduced the effects of IGFBP-3 and peptide in the absence or presence of the anti-CD44 antibody, 5F12. These findings indicate that AChE induces its effect via a mechanism that requires its catalytic activity (Supplementary Tables 3A,B and 4).

## Discussion

We have previously identified an IGF-independent function for IGFBP-3 in blocking HA-CD44 signaling that leads to diminished cell viability in A549 cells^51^. We also found that an IGFBP-3 peptide (^215^-KKGFYKKKQCRPSKGRKR-^232^) is able to bind HA with comparable affinity as the IGFBP-3 protein^51^. In this study, we constructed an IGFBP-3 peptide mutant (K228AR230A) that is unable to bind HA (Table 1, Fig. 1) to serve as a negative control along with the analogous peptide sequences of the rest of the IGFBPs to examine whether they are also able to bind HA and regulate HA-CD44 signaling. WT IGFBP-3 peptide, but not its mutant, bound HA more efficiently than IGFBP-5 or IGFBP-6 peptides while no binding was observed for IGFBP peptides 1, 2, or 4 (Table 1, Fig. 1). The finding that neither IGFBP-1 or IGFBP-4 peptides bound HA was not surprising since they lack the HA binding motif [B(X_7_)B] found in the IGFBP-3 and -5 peptides. While residues Lys227, His228, Asn232, Leu233 and Lys234 necessary for binding heparin were previously identified in IGFBP-2 by site-directed mutagenesis or NMR^34,36,38^, there was a complete lack of binding of IGFBP-2 to HA suggesting that these amino acids are not important for binding HA. Albeit less efficiently than the IGFBP-3 peptide, IGFBP-6 peptide was still able to bind HA despite the absence of the HA binding motif. The presence of the heparin-binding sequence [B-B-B-X-X-B] in this peptide cannot account for its ability to bind HA since this sequence is also preserved in the IGFBP-3 peptide mutant, unable to bind HA.

To examine whether the WT IGFBP-3 peptide operates similarly to the IGFBP-3 protein, we chose to work with a normal human lung cell (HFL1) reported earlier^72^ to be CD44-negative and two human NSCLC cell lines^43^, A549 (p53 WT-genotype) expressing relatively high levels of IGFBP-3 and p53, and H1299 (p53-null genotype) that have undetectable levels of IGFBP-3^73^. Higher levels of HA (3–4 fold) were secreted in the media of A549 and H1299 cells as compared to the amount secreted by HFL1 cells (Fig. 2) and those levels were severely reduced in all three cell lines upon treatment with 4-MU. As HFL1 are CD44-negative^72^, treatment of the cells with the anti-CD44 antibody, 5F12, or the HAS inhibitor, 4-MU, had little effect on cell viability (Fig. 3A). Addition of the IGFBP-3 protein alone or in combination with the antibody, 5F12, or 4-MU, also had very little effect on HFL1 cell viability. Consistent with our previous findings^51^, we found that blocking HA-CD44 interactions in A549 cells with either IGFBP-3, 5F12, or in combination, reduced cell viability to the same extent (Fig. 3B). Similar trends were also observed in the p53-negative cell line, H1299 (Fig. 3C); however, there was only 10–25% decrease in cell viability compared to 50–65% found in A549 cells. These results raised the possibility of links to p53 operative in the mechanism employed by IGFBP-3 in reducing cell viability since H1299 appeared to be more resistant to the effects of blocking HA-CD44 with 5F12, 4-MU, or IGFBP-3 under our conditions. Not surprisingly, the effects on cell viability using the WT IGFBP-3 peptide (Fig. 3D), but not the mutant (Fig. 3E), were comparable to those of the IGFBP-3 protein suggesting that the protein exerts its effects on HA-CD44 signaling via amino acid residues 215–232 of mature IGFBP-3. Consistent with their lack of binding to HA (Fig. 1), IGFBP-1 and -4 peptides showed no effect on cell viability in any of the cell lines (Fig. 3F,H). Despite the presence of residues in the IGFBP-2 peptide shown to bind heparin earlier by site-directed mutagenesis or NMR^34,36,38^, no binding to HA (Fig. 1) and no effect on cell viability (Fig. 3G) was observed, a result similar to those found for IGFBP-1 and -4 peptides. Similar yet more modest effects were observed for both the IGFBP-5 and IGFBP-6 peptides on cell viability as compared to the IGFBP-3 protein or its WT peptide in A549 and H1299 cells (Fig. 3I,J). The relatively more modest inhibition of cell viability correlated with their relatively reduced efficiency of binding to HA (Fig. 1). These results indicate that the IGFBP-5 and -6 peptides also bind HA, are able to disrupt HA-CD44 signaling, albeit to a lesser extent than either the IGFBP-3 protein or its WT peptide, and that their effect is more pronounced in the p53-positive cell line, A549.

Recent reports have suggested that AChE might be a downstream component of p53 that leads to induction of apoptosis^60,61^. AChE was found to be upregulated in response to activation of p53 in apoptotic MCF-7 cells treated with cisplatin. When p53 expression was blocked with siRNA^60^, however, this increase in cisplatin-induced AChE expression was inhibited. Acetylcholine is known to be secreted by lung cancer cells into the extracellular environment, stimulates growth of cancerous cells in lung tumors^65,66^, has mitogenic effects in A549 and the p53-negative H1299 cells, and acts as an autocrine growth factor for human lung cancer^67^ cells promoting their proliferation^67^. Therefore, we hypothesized that one mechanism that might account for the reduced effect of IGFBP-3 on H1299 cell viability might be the lack of p53 expression in this cell line, which leads to inhibiting AChE expression resulting in increased cell viability and decreased apoptosis. In support of this hypothesis, treatment of cells with either the IGFBP-3 protein or WT peptide, the anti-CD44 antibody, 5F12, 4-MU, or in combination resulted in an increased level and activity of AChE in the media of A549 but not in the media of either HFL1 or H1299 cells (Figs. 4, 5). No differences were observed in A549 cells treated with IGFBP-3 protein or peptide, the antibody, the HAS inhibitor, or in combination suggesting that IGFBP-3 exerts its effects on AChE concentrations in a CD44-dependent manner. As expected, since HFL1 are CD44-negative, treatment with the CD44 antibody, 5F12, or inhibition of HAS with 4-MU, had minimal effect on the amount of AChE in the media compared to untreated cells. Western blotting using the same concentration of total protein (15 µL of 600 µg/mL) of the cell supernatants and media (Fig. 6) showed little expression of AChE in H1299 relative to either A549 or HFL1. This relatively low level of AChE and activity (Fig. 5E,F) did not change upon treatment of H1299 cells with IGFBP-3 protein or peptide, 5F12, 4-MU, or in combination despite the abundance of CD44 in these cells. The results were comparable to those obtained with HFL-1 lacking CD44 (Fig. 5A,B). The lack of p53 in H1299 may explain why A549 were more sensitive to the treatments compared to H1299.

**Figure 9:**
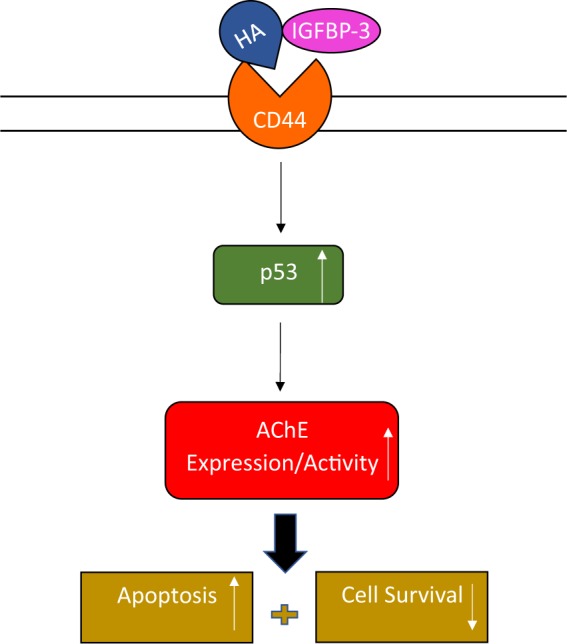
A schematic model summarizing the main results of the current study. IGFBP-3 binds HA and blocks its interaction with CD44 resulting in increased AChE expression and activity in a p53-dependent manner leading to apoptosis and decreased cell survival.

P53 is mutated in ~50% of human cancers and its function as a critical tumor suppressor and in regulation of a large number of genes involved in inhibition of cell proliferation, cell-cycle arrest, apoptosis and senescence, is well-documented^55,56,79^. Several earlier reports showed that the activity of AChE was reduced in lung cancer, likely contributing to lung cancer growth^61,68–70^. Moreover, blocking AChE expression by siRNA or treatment with AChE pharmacological inhibitors, blocked apoptosis^61,71^. Transfection of A549 and HFL1 cells with either p53 siRNA or with AChE siRNA diminished both the levels and activity of AChE in the media (Fig. 7A,B, Supplementary Table 2A,B), while no difference was observed using H1299 cells compared to control. The increased levels and activity of AChE in A549 cells transfected with control siRNA and treated with the IGFBP-3 protein or WT but not mutant peptide, were blocked in A549 cells transfected with either p53 siRNA or AChE siRNA while no difference was observed for either the p53-negative H1299 cell line or the CD44-negative HFL1 cell line subjected to the same treatments. In addition, increased apoptosis and reduced cell viability observed in A549 cells transfected with control siRNA and treated with IGFBP-3 protein or WT peptide (Fig. 8, Supplementary Tables 3AB and 4) were less pronounced in cells transfected with either p53 siRNA or AChE siRNA, but not completely abolished. These results might account for the approximate 10–25% decrease in H1299 cell viability upon treatment of this p53-negative cell line with the IGFBP-3 protein or WT peptide (Fig. 3). Based on our findings, we propose a model (Fig. 9) whereby IGFBP-3 exerts its effect on HA-CD44 signaling via a pathway that depends, in part, on both p53 and AChE. How increased AChE expression and activity in the media leads to decreased cell viability and increased apoptosis in response to IGFBP-3 treatment is currently a mechanism investigated in our laboratory.

## Methods

### Materials

Most of the material used in this study was purchased as we reported earlier^51^. Fluorenylmethyloxycarbonyl (Fmoc) protected amino acids and O-benzotriazolyl-N,N,N′,N′-tetramethyluronium hexafluorophosphate (HBTU) were purchased from Anaspec Inc. Dichloromethane was purchased from Acros Organics. Dimethylformamide (DMF) and HPLC-grade acetonitrile (ACN) were from VWR. Piperidine, triisopropylsilane (TIS), diethyl ether, ethanol, phenol, acetic anhydride, trifluoroacetic acid (TFA), Phosphate Buffer Saline (PBS), nitrocellulose membranes, recombinant human AChE (C1682, UniProt accession ID: C9JD78), AChE Activity Assay Kit (MAK119), HA-biotin (B1557), 4-Methylumbelliferone (4-MU, M1381), streptavidin-horseradish peroxidase (HRP) conjugate, and MISSION human ACHE (esiRNA1, EHU072891), were purchased from Sigma-Aldrich. Rink amide MBHA resin was purchased from Nova Biochem. The AChE specific inhibitor, tacrine hydrochloride and the hyaluronan quantikine ELISA kit (DHYAL0) were purchased from R&D Systems. Recombinant human IGFBP3 protein (YCP1009, UniProt accession ID: P17936) was from Speed BioSystems. CD44 antibody (5F12) (MA5–12394), mouse IgG isotype control, (mIgG), mouse α-tubulin monoclonal antibody (DM1A), 3,3′,5,5′-tetramethylbenzidine, Lipofectamine 2000 Transfection Reagent, amplex acetylcholine/acetlycholinesterase assay kit (A12217), the Halt Protease and Phosphatase Inhibitor Cocktail, and annexin V human ELISA kit (BMS252), were from ThermoFisher. Goat anti-AChE antibody (ab31276), and rabbit anti-Goat IgG H&L (HRP) (ab6741) were purchased from Abcam. Goat anti-rabbit IgG-HRP (sc-2004) was from Santa Cruz Biotechnology. The caspase 3 (cleaved) colorimetric In-Cell ELISA Kit (62218), BCA protein assay kit and the super signal west pico luminol (chemiluminescence) reagent were from Pierce. SignalSilence p53 siRNA I (6231), SignalSilence Control siRNA (Unconjugated, 6568), p53 antibody (9282) were purchased from Cell Signaling Technology.

### Solid phase peptide synthesis

Peptides of IGFBP-1 to -6 (Table 1) corresponding to residues ^215^KKGFYKKKQCRPSKGRKR^232^ of IGFBP-3 along with mutant IGFBP-3 peptide (K228AR230A) were synthesized^51^ on a PS3 synthesizer from Protein Technologies, using rink amide MBHA resin as a solid support on a 0.1 mmole scale. The side chains of Tyr, Glu, Asp, and Ser were protected as the t-butyl derivatives, Lys as t-butyloxycarbonyl (Boc), Gln, Asn, His, and Cys as the trityl (Trt) forms, and Arg as the 2,2,5,7,8-pentamethyl-chroman-6-sulphonyl (Pmc) form. N-alpha-Fmoc protected amino acids were coupled in four-fold excess using HBTU as an activating agent, and 20% piperidine was used for deprotection. The peptides were cleaved from the resin by stirring in a 10 mL cocktail consisting of 5% distilled water, 5% phenol scavenger, 2% TIS, and 88% TFA for 2 h at RT. The peptides were precipitated with cold diethyl ether, filtered, dissolved in 35% ACN/H_2_O and lyophilized. The crude peptides were purified by reversed-phase HPLC on a Phenomenex C18 column (25 cm × 2.2 cm), using 0.1% TFA in water (solvent A) and 0.1% TFA in ACN (solvent B), with a gradient of 10 to 50% B over 2 h at a flow rate of 10 mL/min. Purity was assessed by analytical HPLC on a Phenomenex C18 column (25 cm × 4.6 mm), at 220 nm. The molecular weights were determined using paper spray ionization mass spectrometry. The peptides were dissolved in 10% DMSO and PBS buffer, pH 7.4, to a final concentration of 1 mg/mL.

### Cell culture

HFL1 (ATCC CCL-153) normal, non-transformed and non-tumorigenic human fibroblast cell line, human NSCLC cells (H1299) NCI-H1299 (ATCC CRL-5803), and the human NSCLC cell line, A549 (ATCC CCL-185) were purchased from the American Type Culture Collection (ATCC, Manassas, VA). Cells were seeded as we described earlier^51^ in 25 cm^2^ tissue culture flasks in 5 mL HyClone Dulbecco’s modified Eagle’s medium/nutrient mixture F-12 (DMEM/F12) (GE Healthcare Life Sciences, Pittsburgh, PA), supplemented with 10% Fetalgro bovine growth serum (FBS, RMBIO, Missoula, MT), 50 U/mL penicillin, and 50 U/mL streptomycin (Invitrogen Life Technologies, Carlsbad, CA) and allowed to grow overnight in an incubator at 37 °C, 95% humidity, and 5% CO_2_. The cells were counted using a hemocytometer after trypan blue staining.

### ELISA

Wells of a Nunc MaxiSorp 96-well Flat Bottom plate (ThermoFisher) were coated with 100 µL of 25–200 nM protein/peptide in sample buffer (15 mM Na_2_CO_3_, 50 mM NaHCO_3,_ pH 9.6), and incubated overnight at 4 °C on a shaker to allow the protein/peptide to bind to the plate^51^. Following incubation, the wells were washed 4x with TBST, filled with 400 µL blocking buffer (110 mM KCl, 5 mM NaHCO_3_, 5 mM MgCl_2_, 1 mM EGTA, 0.1 mM CaCl_2_, 20 mM HEPES, 1% BSA, pH 7.4), and incubated overnight at 4 °C on a shaker. After washing 4x with TBST, 100 µL PBS buffer containing the desired concentration of sample were added to each well, and the plate incubated overnight at 4 °C on a shaker to allow interaction of the sample with the protein/peptide bound to the plate. The wells were then washed 4x with TBST before proceeding in one of two ways: 1) If analyzing biotinylated samples, 100 µL streptavidin-HRP conjugate in TBST (1:2500 dilution) were added before incubating for 3 h at RT on a shaker, or 2) when analyzing samples without biotin, 100 µL TBST containing the necessary primary antibody were added at the manufacturer’s recommendation, incubated for 3 h at RT on a shaker, and washed again 4x with TBST. TBST (100 µL) containing the secondary antibody were then added at the manufacturer’s recommendation and incubated for 1 h at RT on a shaker. For either sample type, the plate was then washed 5x with TBST followed by the addition of 100 µL 3,3′,5,5′-tetramethylbenzidine resulting in a blue color change. After incubating at RT for 0.5–15 min, the reaction was stopped with 100 µL 2 M H_2_SO_4_, resulting in a yellow color change, and the absorbance measured at 450 nm. Statistical analysis was determined by the GraphPad Prism 8.3.1 software. Three to five independent experiments were carried out for each assay condition.

### Cell treatment for dot- and western-blotting

Cells were grown in 10% FBS-supplemented media overnight as indicated in 25 cm^2^ flasks (ThermoFisher) then the cell monolayers were incubated in serum-free medium for 12 h. The cells were then treated with 50 nM IGFBP-3 protein or peptides, 4-MU (600 µM), mouse IgG isotype control with no relevant specificity to a target antigen (mIgG, 5 μg/mL), or 5 μg/mL anti-CD44 antibody (5F12) known to be antagonistic towards HA-CD44 molecular interactions. When added in combination, cells were treated for 24 h with 4-MU or for 2 h with the antibodies prior to addition of the IGFBP-3 protein or peptides. This concentration of added IGFBP-3 was chosen to be close to the value measured earlier in the conditioned media of A549 cells^51^. The cells were then allowed to incubate for 48 h and the media collected and analyzed. Attached live cells were harvested and the cell pellet was resuspended in 1 mL lysis buffer consisting of 20 mM Tris/HCl, pH 7.5, 137 mM NaCl, 1% triton X-100, 10% glycerol, 1 mM phenylmethylsulfonyl fluoride (PMSF) and Halt protease and phosphatase inhibitor cocktail (ThermoFisher). Detached apoptotic cells were also harvested and the pellet resuspended in the same lysis buffer. Samples were briefly sonicated, centrifuged and the supernatants stored at −80 °C until further analysis. The protein concentrations were determined using the BCA protein assay kit.

### Dot blotting

After cell treatments, 3 µL of 600 µg/mL total protein of the conditioned media and supernatants prepared from live attached cells and from detached apoptotic cells, were spotted onto a nitrocellulose membrane. As we reported previously^51^, the membrane was allowed to dry then non-specific sites were blocked by soaking the blot in TBST containing 5% BSA in a 10 cm Petri dish for 1 h at RT. The blot was next incubated with goat anti-AChE antibodies in BSA/TBST overnight at RT according to the manufacturer′s instructions. The membrane was then washed three times with TBST (3 × 5 min) then incubated with anti-goat IgG-HRP following the manufacturer’s recommendation for 30 min at RT. The membrane was next washed three times with TBST (3 × 5 min), then once with TBS for 5 min. The amount of AChE on the membrane was detected using super signal west pico luminol (chemiluminescence) reagent, imaged with a Bio-Rad molecular imager, and quantitated using Image J 1.47 v software. Recombinant human AChE and distilled water were used as a positive and negative control, respectively.

### Western blotting

Following methods we reported previously^51^, samples were boiled in 1X SDS, loaded and separated by SDS-PAGE on a 12% gel then transferred to a nitrocellulose membrane. The membrane was blocked in TBST buffer, pH 7.6 containing 5% nonfat milk for 6 h at 4 °C. The membrane was then incubated with the specific primary antibody in the blocking buffer, diluted as specified by the manufacturer at RT overnight with gentle shaking. After washing three times with TBST, the membrane was incubated with a HRP labeled secondary antibody in the blocking buffer, diluted according to the manufacturer’s recommendation. Subsequent to washing three times in TBST, the blots were developed using super signal west pico luminol (chemiluminescence) reagent and imaged with a Bio-Rad molecular imager.

### MTT assay

The MTT reduction assay, used to measure cell viability, was purchased from Sigma-Aldrich and used as we described earlier^51,80^. Cells were seeded in 96-well plates as indicated in 200 μL 10% FBS-supplemented media per well as described above and maintained overnight at 95% humidity and 5% CO_2_. The media from the microplate was then removed and 200 μL serum-free media was added. The cells were allowed to incubate for 12 h. The cells were then treated with 50 nM IGFBP-3 protein or peptides, control mIgG (5 μg/mL), 600 µM 4-MU, or with 5 μg/mL CD44 antibody (5F12) for 48 h. When added in combination, cells were pretreated with the antibodies for 2 h or with 4-MU for 48 h prior to addition of the protein or peptides. Certain wells containing only media with DMSO were used as a negative control. In each well, the final concentration of DMSO never exceeded 0.1%. Subsequent to a 48 h incubation, the cells were incubated for 4 h with MTT (0.5 mg/mL) then the media was carefully removed. Due to sensitivity of the MTT reagent, the MTT assays were carried out in the dark. DMSO (100 μL) was added to dissolve the formazan crystals then the absorbance was measured at 570 nm in a plate reader. Untreated cells were used as a positive control and as a negative control, DMSO alone was used. Statistical analysis was conducted using GraphPad Prism version 8.3.1 for Windows. Compared with the control, significant values were considered at p < 0.05 and more significant values at p < 0.01.

### Apoptosis assays

For the caspase 3 (cleaved) colorimetric assays, activated (cleaved) caspase 3 and tubulin, were simultaneously measured in triplicate in whole cells by a colorimetric in cell ELISA assay (ThermoFisher) using 96-well microplates as we previously described^80,81^. Cells were plated per well and incubated overnight at 37 °C in 5% CO_2_. Cells were treated as described above for the MTT assay then subsequently fixed with 4% formaldehyde, permeabilized according to the manufacturer’s instructions, and incubated with primary antibodies overnight at 4 °C. For the annexin V human ELISA kit, annexin^82^ is detected in the cell culture medium by a matched antibody pair and quantitated using a standard curve for human Annexin V according to the instructions by the manufacturer. For both methods, HRP conjugates were added next and incubated at RT for 30 min. Subsequent to washing the plates, the TMB substrate was added to each well and incubated in the dark at RT. The reaction was typically stopped after 15 min with a TMB stop solution when the blue color became apparent. The absorbance was then measured at 450 nm within 30 min after the reaction was stopped. Control cells were treated with a 0.1% DMSO vehicle control and contained all the reagents except the primary antibodies. The average of all replicate nonspecific background signal controls from each condition was subtracted then the average absorbance at 450 nm for each condition was calculated.

### AChE activity

AChE activity of the conditioned media and cell supernatants was assayed by the Ellman method using the AChE Activity Assay Kit (MAK119) and according to methods previously reported^67,77^. This assay measures a colorimetric (412 nm) product formed from thiocholine, produced by AChE, which reacts with 5,5′-dithiobis (2-nitrobenzoic acid). One unit of AChE is the amount of enzyme that catalyzes the production of 1.0 µmole of thiocholine per min at pH 8 at 37 °C. The colorimetric product is proportional to the AChE activity present.

### SiRNA transfection

Transfections were carried out according to methods reported earlier^60^. The day before transfection, cells were seeded at a density of 2 × 10^4^ in 96-well microplates. Control siRNA, p53 siRNA, or AChE siRNA was mixed with Lipofectamine 2000 transfection reagent diluted in Opti-MEM Medium (ThermoFisher) for 20 min at RT. The mixtures were then added to the cells to a final concentration of 100 nM for each siRNA without and with specific added treatments as indicated. The cells were then allowed to incubate from 24 to 72 h at 37 °C and subsequently used in different assays. Cells exposed to Lipofectamine 2000 alone were used as a mock control. AChE activity was assayed in the media. Cells collected by trypsinization at the different intervals after transfection along with the media were used for dot and Western blotting, while cell viability and apoptosis were measured as described above. Each measurement represents the mean ± S.D. of three independent experiments, each performed in triplicate.

### Statistical analysis

All experiments were performed in triplicate and repeated a minimum of three times. Statistical values are expressed as the mean ± Standard Deviation (SD). The Mann–Whitney or Kruskal–Wallis (ANOVA) tests were used to evaluate the statistical differences. All the statistical tests were two-sided and a P value of <0.05 was considered statistically significant in all cases. GraphPad Prism (GraphPad Software, 8.3.1) was employed for the statistical analysis.

## Supplementary information

Supplementary information.
