# Supplementary material for: IGFBP-3 Blocks Hyaluronan-CD44 Signaling, Leading to Increased Acetylcholinesterase Levels in A549 Cell Media and Apoptosis in a p53-Dependent Manner
Source: Sci Rep. 2020 Mar 19;10:5083. doi: 10.1038/s41598-020-61743-3 (PMC7081274; doi:10.1038/s41598-020-61743-3)
Supplement: Supplementary file 1 — Supplementary information. [file 41598_2020_61743_MOESM1_ESM.pdf]

Evans Manuscript Supplementary Information

IGFBP-3 Blocks Hyaluronan-CD44 Signaling, Leading to Increased Acetylcholinesterase Levels in A549 Cell Media and Apoptosis in a p53-Dependent Manner

Deanna Price, Robert Muterspaugh, Bradley Clegg, Asana Williams, Alexis Stephens, Jeffrey Guthrie, Deborah Heyl, and Hedeel Guy Evans*

Supplementary Table 2A. Fold Change of the Amount of AChE in the Media Relative to Control.

Cells (2 x 10^4) were transfected with 100 nM siRNA for 24h then treated with either 50 nM of the IGFBP-3 protein or peptides, 600 µM 4-MU, the CD44 antibody (5F12, 5 µg/mL), or in combination for an additional 48h. After cell treatments, 3 µL of 600 µg/mL total protein of the conditioned media were spotted onto a nitrocellulose membrane. The blots were incubated with goat anti-AChE antibodies and the amount of AChE on the membrane was detected using super signal west pico luminol (chemiluminescence) reagent, imaged with a Bio-Rad molecular imager, and quantitated using Image J (Methods). The results are expressed as the mean ± S.D. of three independent experiments, each performed in triplicate.

Treatment	A549	H1299	HFL1
Control siRNA	1.0 ± 0.16	1.0 ± 0.12	1.0 ± 0.14
P53 siRNA	0.19 ± 0.04	1.1 ± 0.17	0.19 ± 0.03
AChE siRNA	0.18 ± 0.03	0.96 ± 0.14	0.15 ± 0.02
IGFBP-3 Protein + Control siRNA	3.8 ± 0.61	0.91 ± 0.13	1.1 ± 0.15
IGFBP-3 Peptide + Control siRNA	3.6 ± 0.56	1.0 ± 0.15	1.1 ± 0.17
IGFBP-3 Mutant Peptide + Control siRNA	0.94 ± 0.15	0.99 ± 0.16	0.98 ± 0.11
IGFBP-3 Protein + p53 siRNA	0.21 ± 0.04	0.94 ± 0.16	0.16 ± 0.03
IGFBP-3 Peptide + p53 siRNA	0.20 ± 0.03	0.98 ± 0.15	0.15 ± 0.02
IGFBP-3 Mutant Peptide + p53 siRNA	0.19 ± 0.03	1.1 ± 0.16	0.19 ± 0.03
IGFBP-3 Protein + AChE siRNA	0.17 ± 0.03	0.97 ± 0.14	0.18 ± 0.03
IGFBP-3 Peptide + AChE siRNA	0.15 ± 0.03	0.95 ± 0.13	0.17 ± 0.02
IGFBP-3 Mutant Peptide + AChE siRNA	0.16 ± 0.02	0.93 ± 0.15	0.15 ± 0.03
5F12 + Control siRNA	3.9 ± 0.57	1.2 ± 0.18	1.1 ± 0.17
5F12 + IGFBP-3 Protein + Control siRNA	4.0 ± 0.62	0.98 ± 0.15	1.1 ± 0.15
5F12 + IGFBP-3 Peptide + Control siRNA	3.6 ± 0.43	1.1 ± 0.16	1.2 ± 0.16
5F12 + IGFBP-3 Mutant Peptide + Control siRNA	3.8 ± 0.54	0.99 ± 0.15	1.1 ± 0.18
5F12 + p53 siRNA	0.21 ± 0.04	1.1 ± 0.16	0.17 ± 0.02
5F12 + IGFBP-3 Protein + p53 siRNA	0.20 ± 0.02	1.1 ± 0.15	0.19 ± 0.03
5F12 + IGFBP-3 Peptide + p53 siRNA	0.19 ± 0.04	1.2 ± 0.18	0.18 ± 0.02
5F12 + IGFBP-3 Mutant Peptide + p53 siRNA	0.18 ± 0.03	0.99 ± 0.16	0.15 ± 0.02
5F12 + AChE siRNA	0.17 ± 0.02	0.97 ± 0.14	0.14 ± 0.01
5F12 + IGFBP-3 Protein + AChE siRNA	0.18 ± 0.02	0.96 ± 0.15	0.16 ± 0.02
5F12 + IGFBP-3 Peptide + AChE siRNA	0.19 ± 0.03	0.98 ± 0.14	0.12 ± 0.02
5F12 + IGFBP-3 Mutant Peptide + AChE siRNA	0.18 ± 0.03	0.94 ± 0.12	0.16 ± 0.03
4-MU + Control siRNA	3.6 ± 0.57	1.1 ± 0.16	1.1 ± 0.16
4-MU + IGFBP-3 Protein + Control siRNA	3.6 ± 0.42	1.0 ± 0.12	1.1 ± 0.17
4-MU + IGFBP-3 Peptide + Control siRNA	3.7 ± 0.45	0.98 ± 0.16	1.1 ± 0.16
4-MU + IGFBP-3 Mutant Peptide + Control siRNA	4.0 ± 0.59	0.98 ± 0.13	1.0 ± 0.14
4-MU + p53 siRNA	0.20 ± 0.02	0.99 ± 0.16	0.16 ± 0.02
4-MU + IGFBP-3 Protein + p53 siRNA	0.18 ± 0.03	1.1 ± 0.16	0.19 ± 0.04
4-MU + IGFBP-3 Peptide + p53 siRNA	0.17 ± 0.02	1.0 ± 0.15	0.17 ± 0.02
4-MU + IGFBP-3 Mutant Peptide + p53 siRNA	0.19 ± 0.03	1.1 ± 0.16	0.16 ± 0.02
4-MU + AChE siRNA	0.16 ± 0.02	0.93 ± 0.14	0.12 ± 0.01
4-MU + IGFBP-3 Protein + AChE siRNA	0.19 ± 0.03	0.94 ± 0.15	0.13 ± 0.02
4-MU + IGFBP-3 Peptide + AChE siRNA	0.17 ± 0.02	0.96 ± 0.12	0.11 ± 0.02
4-MU + IGFBP-3 Mutant Peptide + AChE siRNA	0.16 ± 0.02	0.96 ± 0.13	0.15 ± 0.03

SupplementaryTable 2B. AChE Activity (nmol/min/mg) in the Media

Cells (2 x 10^4) were transfected with 100 nM siRNA for 24h then treated with either 50 nM of the IGFBP-3 protein or peptides, 600 µM 4-MU, the CD44 antibody (5F12, 5 µg/mL), or in combination for an additional 48h. After cell treatments, 3 µL of 600 µg/mL total protein of the conditioned media were assayed for AChE activity (Methods). The results are expressed as the mean ± S.D. of three independent experiments, each performed in triplicate.

Treatment	A549	H1299	HFL1
Control siRNA	33 ± 4.0	5 ± 0.6	13 ± 5.0
P53 siRNA	8 ± 1.3	3 ± 0.4	7 ± 1.1
AChE siRNA	7 ± 1.2	3 ± 0.3	6 ± 0.9
IGFBP-3 Protein + Control siRNA	138 ± 23	5 ± 0.5	17 ± 3.0
IGFBP-3 Peptide + Control siRNA	136 ± 21	5 ± 0.5	18 ± 5.0
IGFBP-3 Mutant Peptide + Control siRNA	29 ± 5.0	4 ± 0.6	12 ± 2.0
IGFBP-3 Protein + p53 siRNA	6 ± 1.2	2 ± 0.3	7 ± 1.3
IGFBP-3 Peptide + p53 siRNA	7 ± 1.3	3 ± 0.6	5 ± 0.6
IGFBP-3 Mutant Peptide + p53 siRNA	8 ± 1.2	4 ± 0.3	6 ± 0.9
IGFBP-3 Protein + AChE siRNA	6 ± 1.1	3 ± 0.4	5 ± 0.9
IGFBP-3 Peptide + AChE siRNA	7 ± 1.2	3 ± 0.3	4 ± 0.5
IGFBP-3 Mutant Peptide + AChE siRNA	6 ± 1.2	4 ± 0.4	5 ± 0.8
5F12 + Control siRNA	148 ± 24	5 ± 0.7	17 ± 3.0
5F12 + IGFBP-3 Protein + Control siRNA	141 ± 26	5 ± 0.6	18 ± 3.0
5F12 + IGFBP-3 Peptide + Control siRNA	145 ± 27	5 ± 0.5	16 ± 2.0
5F12 + IGFBP-3 Mutant Peptide + Control siRNA	139 ± 24	5 ± 0.6	17 ± 3.0
5F12 + p53 siRNA	9 ± 1.4	3 ± 0.3	6 ± 1.0
5F12 + IGFBP-3 Protein + p53 siRNA	8 ± 1.5	4 ± 0.5	7 ± 1.2
5F12 + IGFBP-3 Peptide + p53 siRNA	8 ± 1.4	4 ± 0.6	6 ± 1.1
5F12 + IGFBP-3 Mutant Peptide + p53 siRNA	7 ± 1.2	3 ± 0.3	5 ± 0.8
5F12 + AChE siRNA	6 ± 0.9	3 ± 0.6	7 ± 1.1
5F12 + IGFBP-3 Protein + AChE siRNA	6 ± 1.1	4 ± 0.2	6 ± 0.8
5F12 + IGFBP-3 Peptide + AChE siRNA	5 ± 0.8	4 ± 0.3	5 ± 1.0
5F12 + IGFBP-3 Mutant Peptide + AChE siRNA	6 ± 1.2	3 ± 0.4	6 ± 0.9
4-MU + Control siRNA	143 ± 21	4 ± 0.6	16 ± 2.0
4-MU + IGFBP-3 Protein + Control siRNA	138 ± 23	4 ± 0.5	17 ± 2.0
4-MU + IGFBP-3 Peptide + Control siRNA	149 ± 28	3 ± 0.2	18 ± 3.0
4-MU + IGFBP-3 Mutant Peptide + Control siRNA	142 ± 23	4 ± 0.4	16 ± 2.0
4-MU + p53 siRNA	6 ± 0.8	2 ± 0.6	7 ± 1.2
4-MU + IGFBP-3 Protein + p53 siRNA	9 ± 1.4	5 ± 0.6	6 ± 0.9
4-MU + IGFBP-3 Peptide + p53 siRNA	7 ± 1.3	5 ± 0.5	5 ± 0.8
4-MU + IGFBP-3 Mutant Peptide + p53 siRNA	8 ± 1.2	4 ± 0.6	7 ± 0.9
4-MU + AChE siRNA	6 ± 1.1	4 ± 0.4	6 ± 1.0
4-MU + IGFBP-3 Protein + AChE siRNA	9 ± 1.3	5 ± 0.5	8 ± 1.2
4-MU + IGFBP-3 Peptide + AChE siRNA	7 ± 1.3	3 ± 0.4	7 ± 1.1
4-MU + IGFBP-3 Mutant Peptide + AChE siRNA	9 ± 1.2	5 ± 0.5	8 ± 0.9

Supplementary Table 3A. Apoptosis (Caspase 3 assay, fold change relative to cells treated with control siRNA)

Cells plated in a 96-well plate (2 x 10^4 cells/well in 200 µL medium) were transfected with 100 nM siRNA for 24h then treated with 50 nM IGFBP-3 protein or peptides in the absence or presence of the CD44 antibody (5F12, 5 µg/mL), 600 µM 4-MU, or 1 µM tacrine for an additional 48h. After cell treatment, apoptosis was measured as described in Methods. The results are expressed as means ± S.D. of three independent experiments, each performed in triplicate.

Treatment	A549	H1299	HFL1
Control siRNA	1.0 ± 0.14	1.0 ± 0.12	1.0 ± 0.11
P53 siRNA	0.52 ± 0.07	1.1 ± 0.14	0.51 ± 0.09
AChE siRNA	0.67 ± 0.09	0.99 ± 0.13	0.62 ± 0.08
IGFBP-3 Protein + Control siRNA	1.6 ± 0.31	1.2 ± 0.17	1.1 ± 0.15
IGFBP-3 Peptide + Control siRNA	1.7 ± 0.29	1.2 ± 0.16	1.1 ± 0.16
IGFBP-3 Mutant Peptide + Control siRNA	0.98 ± 0.15	0.99 ± 0.20	0.94 ± 0.15
IGFBP-3 Protein + Control siRNA + Tacrine	0.84 ± 0.17	1.2 ± 0.14	0.64 ± 0.10
IGFBP-3 Peptide + Control siRNA + Tacrine	0.85 ± 0.15	1.3 ± 0.15	0.63 ± 0.08
IGFBP-3 Mutant Peptide + Control siRNA + Tacrine	0.68 ± 0.10	0.91 ± 0.12	0.60 ± 0.10
IGFBP-3 Protein + p53 siRNA	0.69 ± 0.14	1.2 ± 0.18	0.53 ± 0.10
IGFBP-3 Peptide + p53 siRNA	0.70 ± 0.15	1.1 ± 0.18	0.54 ± 0.13
IGFBP-3 Mutant Peptide + p53 siRNA	0.54 ± 0.08	0.99 ± 0.16	0.49 ± 0.08
IGFBP-3 Protein + AChE siRNA	0.89 ± 0.16	1.2 ± 0.17	0.63 ± 0.10
IGFBP-3 Peptide + AChE siRNA	0.82 ± 0.17	1.1 ± 0.16	0.64 ± 0.09
IGFBP-3 Mutant Peptide + AChE siRNA	0.66 ± 0.11	0.90 ± 0.14	0.59 ± 0.07
5F12 + Control siRNA	1.6 ± 0.25	1.1 ± 0.17	1.1 ± 0.15
5F12 + Control siRNA + Tacrine	0.88 ± 0.14	1.2 ± 0.13	0.65 ± 0.09
5F12 + IGFBP-3 Protein + Control siRNA	1.7 ± 0.37	1.1 ± 0.12	1.2 ± 0.13
5F12 + IGFBP-3 Peptide + Control siRNA	1.6 ± 0.25	1.2 ± 0.11	1.1 ± 0.15
5F12 + IGFBP-3 Mutant Peptide + Control siRNA	1.8 ± 0.30	1.1 ± 0.16	1.2 ± 0.10
5F12 + IGFBP-3 Protein + Control siRNA + Tacrine	0.84 ± 0.15	1.1 ± 0.17	0.63 ± 0.10
5F12 + IGFBP-3 Peptide + Control siRNA + Tacrine	0.83 ± 0.14	1.2 ± 0.17	0.62 ± 0.08
5F12 + IGFBP-3 Mutant Peptide + Control siRNA + Tacrine	0.85 ± 0.13	1.2 ± 0.15	0.64 ± 0.08
5F12 + p53 siRNA	0.71 ± 0.13	1.1 ± 0.14	0.53 ± 0.12
5F12 + IGFBP-3 Protein + p53 siRNA	0.69 ± 0.12	1.2 ± 0.15	0.54 ± 0.14
5F12 + IGFBP-3 Peptide + p53 siRNA	0.66 ± 0.11	1.1 ± 0.16	0.53 ± 0.11
5F12 + IGFBP-3 Mutant Peptide + p53 siRNA	0.64 ± 0.10	1.1 ± 0.15	0.55 ± 0.09
5F12 + AChE siRNA	0.88 ± 0.16	1.1 ± 0.14	0.63 ± 0.07
5F12 + IGFBP-3 Protein + AChE siRNA	0.82 ± 0.14	1.2 ± 0.15	0.65 ± 0.06
5F12 + IGFBP-3 Peptide + AChE siRNA	0.84 ± 0.13	1.1 ± 0.17	0.64 ± 0.08
5F12 + IGFBP-3 Mutant Peptide + AChE siRNA	0.85 ± 0.15	1.2 ± 0.16	0.63 ± 0.11
4-MU + Control siRNA	1.5 ± 0.22	1.2 ± 0.16	1.0 ± 0.13
4-MU + IGFBP-3 Protein + Control siRNA	1.6 ± 0.27	1.1 ± 0.13	1.1 ± 0.14
4-MU + IGFBP-3 Peptide + Control siRNA	1.8 ± 0.28	1.1 ± 0.15	1.2 ± 0.11
4-MU + IGFBP-3 Mutant Peptide + Control siRNA	1.6 ± 0.31	1.2 ± 0.14	1.1 ± 0.12
4-MU + p53 siRNA	0.81 ± 0.14	1.1 ± 0.11	0.63 ± 0.11
4-MU + IGFBP-3 Protein + p53 siRNA	0.65 ± 0.09	1.1 ± 0.13	0.62 ± 0.09
4-MU + IGFBP-3 Peptide + p53 siRNA	0.70 ± 0.14	1.1 ± 0.12	0.59 ± 0.08
4-MU + IGFBP-3 Mutant Peptide + p53 siRNA	0.61 ± 0.08	1.2 ± 0.14	0.58 ± 0.10
4-MU + AChE siRNA	0.83 ± 0.13	1.2 ± 0.16	0.67 ± 0.09
4-MU + IGFBP-3 Protein + AChE siRNA	0.86 ± 0.16	1.2 ± 0.12	0.69 ± 0.07
4-MU + IGFBP-3 Peptide + AChE siRNA	0.81 ± 0.10	1.2 ± 0.14	0.62 ± 0.06
4-MU + IGFBP-3 Mutant Peptide + AChE siRNA	0.84 ± 0.13	1.2 ± 0.13	0.61 ± 0.08

Supplementary Table 3B. Apoptosis (Annexin V assay, fold change relative to cells treated with control siRNA)

Cells plated in a 96-well plate (2 x 10^4 cells/well in 200 µL medium) were transfected with 100 nM siRNA for 24h then treated with 50 nM IGFBP-3 protein or peptides in the absence or presence of the CD44 antibody (5F12, 5 µg/mL), or 600 µM 4-MU for an additional 48h. After cell treatment, apoptosis was measured as described in Methods. The results are expressed as means ± S.D. of three independent experiments, each performed in triplicate.

Treatment	A549	H1299	HFL1
Control siRNA	1.0 ± 0.13	1.0 ± 0.11	1.0 ± 0.14
P53 siRNA	0.50 ± 0.06	1.1 ± 0.12	0.50 ± 0.08
AChE siRNA	0.68 ± 0.07	0.99 ± 0.12	0.64 ± 0.10
IGFBP-3 Protein + Control siRNA	1.5 ± 0.21	1.1 ± 0.15	1.0 ± 0.15
IGFBP-3 Peptide + Control siRNA	1.6 ± 0.19	1.2 ± 0.14	1.1 ± 0.14
IGFBP-3 Mutant Peptide + Control siRNA	0.99 ± 0.14	0.97 ± 0.17	0.95 ± 0.15
IGFBP-3 Protein + p53 siRNA	0.67 ± 0.13	1.1 ± 0.16	0.55 ± 0.10
IGFBP-3 Peptide + p53 siRNA	0.73 ± 0.16	1.1 ± 0.14	0.52 ± 0.11
IGFBP-3 Mutant Peptide + p53 siRNA	0.52 ± 0.07	0.96 ± 0.13	0.47 ± 0.08
IGFBP-3 Protein + AChE siRNA	0.86 ± 0.18	1.2 ± 0.15	0.61 ± 0.11
IGFBP-3 Peptide + AChE siRNA	0.79 ± 0.15	1.1 ± 0.14	0.63 ± 0.11
IGFBP-3 Mutant Peptide + AChE siRNA	0.69 ± 0.12	0.93 ± 0.15	0.61 ± 0.07
5F12 + Control siRNA	1.8 ± 0.27	1.0 ± 0.16	1.1 ± 0.18
5F12 + IGFBP-3 Protein + Control siRNA	1.7 ± 0.27	1.1 ± 0.14	1.2 ± 0.11
5F12 + IGFBP-3 Peptide + Control siRNA	1.5 ± 0.22	1.2 ± 0.15	1.1 ± 0.16
5F12 + IGFBP-3 Mutant Peptide + Control siRNA	1.8 ± 0.20	1.1 ± 0.17	1.2 ± 0.13
5F12 + p53 siRNA	0.72 ± 0.14	1.1 ± 0.16	0.52 ± 0.12
5F12 + IGFBP-3 Protein + p53 siRNA	0.64 ± 0.12	1.2 ± 0.12	0.51 ± 0.14
5F12 + IGFBP-3 Peptide + p53 siRNA	0.65 ± 0.11	1.2 ± 0.16	0.54 ± 0.11
5F12 + IGFBP-3 Mutant Peptide + p53 siRNA	0.66 ± 0.13	1.1 ± 0.14	0.54 ± 0.09
5F12 + AChE siRNA	0.87 ± 0.16	1.1 ± 0.12	0.61 ± 0.07
5F12 + IGFBP-3 Protein + AChE siRNA	0.83 ± 0.12	1.2 ± 0.13	0.62 ± 0.04
5F12 + IGFBP-3 Peptide + AChE siRNA	0.86 ± 0.13	1.1 ± 0.14	0.64 ± 0.10
5F12 + IGFBP-3 Mutant Peptide + AChE siRNA	0.83 ± 0.14	1.1 ± 0.16	0.63 ± 0.12
4-MU + Control siRNA	1.7 ± 0.21	1.2 ± 0.14	1.2 ± 0.14
4-MU + IGFBP-3 Protein + Control siRNA	1.5 ± 0.17	1.2 ± 0.16	1.1 ± 0.12
4-MU + IGFBP-3 Peptide + Control siRNA	1.7 ± 0.22	1.1 ± 0.12	1.1 ± 0.10
4-MU + IGFBP-3 Mutant Peptide + Control siRNA	1.8 ± 0.41	1.2 ± 0.12	1.1 ± 0.11
4-MU + p53 siRNA	0.78 ± 0.14	1.1 ± 0.10	0.64 ± 0.12
4-MU + IGFBP-3 Protein + p53 siRNA	0.66 ± 0.11	1.2 ± 0.12	0.64 ± 0.11
4-MU + IGFBP-3 Peptide + p53 siRNA	0.65 ± 0.13	1.1 ± 0.11	0.62 ± 0.09
4-MU + IGFBP-3 Mutant Peptide + p53 siRNA	0.59 ± 0.08	1.1 ± 0.14	0.60 ± 0.12
4-MU + AChE siRNA	0.85 ± 0.13	1.2 ± 0.14	0.64 ± 0.09
4-MU + IGFBP-3 Protein + AChE siRNA	0.84 ± 0.12	1.1 ± 0.14	0.70 ± 0.09
4-MU + IGFBP-3 Peptide + AChE siRNA	0.83 ± 0.11	1.1 ± 0.14	0.63 ± 0.05
4-MU + IGFBP-3 Mutant Peptide + AChE siRNA	0.82 ± 0.13	1.2 ± 0.10	0.62 ± 0.09

Supplementary Table 4. Cell viability (fold change relative to cells treated with control siRNA)

Cells plated in a 96-well plate (2 x 10^4 cells/well in 200 µL medium) were transfected with 100 nM siRNA for 24h then treated with either 50 nM of the IGFBP-3 protein or peptides, 600 µM 4-MU, the CD44 antibody (5F12, 5 µg/mL added alone or 2h prior to addition of IGFBP-3 and/or peptides), or 1 µM tacrine for an additional 48h. After cell treatment, cell viability was measured (Methods). The results are expressed as means ± S.D. of three independent experiments, each performed in triplicate.

Treatment	A549	H1299	HFL1
Control siRNA	1.0 ± 0.12	1.0 ± 0.10	1.0 ± 0.13
P53 siRNA	1.6 ± 0.30	0.99 ± 0.16	1.6 ± 0.22
AChE siRNA	1.4 ± 0.25	1.1 ± 0.11	1.5 ± 0.21
IGFBP-3 Protein + Control siRNA	0.42 ± 0.07	0.86 ± 0.11	0.95 ± 0.13
IGFBP-3 Peptide + Control siRNA	0.46 ± 0.08	0.88 ± 0.13	0.96 ± 0.14
IGFBP-3 Mutant Peptide + Control siRNA	0.98 ± 0.10	1.05 ± 0.11	1.1 ± 0.15
IGFBP-3 Protein + Control siRNA + Tacrine	1.3 ± 0.21	0.86 ± 0.12	1.4 ± 0.22
IGFBP-3 Peptide + Control siRNA + Tacrine	1.2 ± 0.22	0.88 ± 0.11	1.5 ± 0.25
IGFBP-3 Mutant Peptide + Control siRNA + Tacrine	1.4 ± 0.21	1.06 ± 0.18	1.6 ± 0.19
IGFBP-3 Protein + p53 siRNA	1.4 ± 0.21	0.84 ± 0.14	1.5 ± 0.18
IGFBP-3 Peptide + p53 siRNA	1.4 ± 0.23	0.87 ± 0.11	1.6 ± 0.16
IGFBP-3 Mutant Peptide + p53 siRNA	1.6 ± 0.35	0.99 ± 0.14	1.6 ± 0.21
IGFBP-3 Protein + AChE siRNA	1.3 ± 0.19	0.83 ± 0.15	1.5 ± 0.21
IGFBP-3 Peptide + AChE siRNA	1.2 ± 0.18	0.84 ± 0.14	1.6 ± 0.18
IGFBP-3 Mutant Peptide + AChE siRNA	1.4 ± 0.20	1.1 ± 0.15	1.5 ± 0.16
5F12 + Control siRNA	0.45 ± 0.06	0.86 ± 0.13	0.93 ± 0.12
5F12 + Control siRNA + Tacrine	1.2 ± 0.19	0.85 ± 0.12	1.6 ± 0.24
5F12 + IGFBP-3 Protein + Control siRNA	0.45 ± 0.08	0.88 ± 0.10	0.91 ± 0.13
5F12 + IGFBP-3 Peptide + Control siRNA	0.47 ± 0.09	0.85 ± 0.15	0.92 ± 0.13
5F12 + IGFBP-3 Mutant Peptide + Control siRNA	0.48 ± 0.10	0.89 ± 0.16	0.90 ± 0.14
5F12 + IGFBP-3 Protein + Control siRNA + Tacrine	1.3 ± 0.20	0.88 ± 0.12	1.4 ± 0.20
5F12 + IGFBP-3 Peptide + Control siRNA + Tacrine	1.2 ± 0.21	0.82 ± 0.13	1.5 ± 0.15
5F12 + IGFBP-3 Mutant Peptide + Control siRNA + Tacrine	1.3 ± 0.19	0.85 ± 0.11	1.6 ± 0.22
5F12 + p53 siRNA	1.4 ± 0.22	0.87 ± 0.10	1.5 ± 0.25
5F12 + IGFBP-3 Protein + p53 siRNA	1.5 ± 0.19	0.88 ± 0.11	1.6 ± 0.20
5F12 + IGFBP-3 Peptide + p53 siRNA	1.4 ± 0.20	0.85 ± 0.14	1.5 ± 0.15
5F12 + IGFBP-3 Mutant Peptide + p53 siRNA	1.5 ± 0.19	0.86 ± 0.13	1.5 ± 0.17
5F12 + AChE siRNA	1.3 ± 0.20	0.85 ± 0.12	1.6 ± 0.19
5F12 + IGFBP-3 Protein + AChE siRNA	1.2 ± 0.21	0.86 ± 0.11	1.4 ± 0.24
5F12 + IGFBP-3 Peptide + AChE siRNA	1.3 ± 0.19	0.85 ± 0.10	1.5 ± 0.15
5F12 + IGFBP-3 Mutant Peptide + AChE siRNA	1.2 ± 0.17	0.82 ± 0.12	1.5 ± 0.23
4-MU + Control siRNA	0.47 ± 0.07	0.87 ± 0.14	0.91 ± 0.11
4-MU + IGFBP-3 Protein + Control siRNA	0.46 ± 0.07	0.86 ± 0.12	0.89 ± 0.10
4-MU + IGFBP-3 Peptide + Control siRNA	0.45 ± 0.11	0.88 ± 0.16	0.93 ± 0.15
4-MU + IGFBP-3 Mutant Peptide + Control siRNA	0.46 ± 0.09	0.85 ± 0.14	0.87 ± 0.12
4-MU + p53 siRNA	1.2 ± 0.17	0.86 ± 0.12	1.6 ± 0.22
4-MU + IGFBP-3 Protein + p53 siRNA	1.3 ± 0.18	0.86 ± 0.12	1.8 ± 0.18
4-MU + IGFBP-3 Peptide + p53 siRNA	1.5 ± 0.18	0.87 ± 0.13	1.7 ± 0.17
4-MU + IGFBP-3 Mutant Peptide + p53 siRNA	1.6 ± 0.20	0.88 ± 0.14	1.6 ± 0.17
4-MU + AChE siRNA	1.2 ± 0.18	0.83 ± 0.11	1.5 ± 0.16
4-MU + IGFBP-3 Protein + AChE siRNA	1.1 ± 0.16	0.84 ± 0.13	1.7 ± 0.21
4-MU + IGFBP-3 Peptide + AChE siRNA	1.2 ± 0.17	0.88 ± 0.15	1.4 ± 0.16
4-MU + IGFBP-3 Mutant Peptide + AChE siRNA	1.1 ± 0.15	0.79 ± 0.11	1.4 ± 0.18
